# Potential Mechanobiological Consequences of Injectable Aesthetic Procedures: Dermal Fillers, Botulinum Toxin, ECM Dynamics, Lymphovascular Drainage, and the Concept of Mechanical Silence

**DOI:** 10.3390/bioengineering13101219

**Published:** 2026-10-19

**Authors:** Wonseok Choi, Kyu-Ho Yi, Olena Sydorchuk

**Affiliations:** 1 V Plastic Surgery, Daegu 41940, Republic of Korea; plastsurgeon@naver.com; 2 Division in Anatomy and Developmental Biology, Department of Oral Biology, Human Identification Research Institute, BK21 FOUR Project, Yonsei University College of Dentistry, Seoul 03722, Republic of Korea; 3 Aesthefora Inc., Seoul 04364, Republic of Korea

**Keywords:** dermal filler, botulinum toxin, mechanotransduction, extracellular matrix, lymphatic drainage, vascular circulation, pressure injury, facial aging, mechanical silence, fibroblast

## Abstract

Injectable aesthetic procedures are commonly discussed in terms of volumization, muscle relaxation, wrinkle reduction, and adverse events, whereas their potential effects on the dynamic mechanical environment of facial soft tissues have received less attention. The dermis is a mechanosensitive, viscoelastic tissue in which fibroblasts, extracellular matrix (ECM), interstitial fluid, vascular and lymphatic physiology, and repeated facial motion interact. Established evidence demonstrates that appropriately used hyaluronic acid fillers can restore structural support and stimulate collagen remodeling, while botulinum toxin reduces muscle contraction and dynamic rhytides. By contrast, direct clinical evidence that injectable procedures produce sustained abnormalities in tissue mechanics, lymphatic pumping, or mechanotransductive signaling remains limited. This narrative review integrates established clinical evidence, mechanistic evidence from adjacent fields, and hypothesis-generating interpretations to propose the concept of injectable procedure-related “mechanical silence”: a localized reduction or distortion of physiological tissue deformation, fluid exchange, and cell–matrix mechanical signaling. The concept is not presented as a demonstrated adverse effect or as evidence that fillers or botulinum toxin accelerate aging. Rather, it provides a testable framework for investigating whether selected patterns of excessive volume, altered local stiffness, persistent edema, or marked reduction in muscle-driven tissue motion are associated with measurable changes in tissue biomechanics. Pressure-injury biology is discussed only as a conceptual example of the consequences of sustained deformation and impaired tissue mobility and is not considered mechanically or clinically equivalent to aesthetic injectable treatment. Future studies should combine structural imaging with quantitative assessment of tissue stiffness, deformation and recoil, filler burden, lymphatic transit, perfusion, muscle excursion, and mechanotransductive biomarkers to determine whether the proposed framework has clinical relevance.

## 1. Introduction

Dermal fillers and botulinum toxin are among the most frequently used minimally invasive aesthetic interventions. Their conventional clinical roles are straightforward: fillers replace or redistribute volume, improve contour, and support soft tissue; botulinum toxin reduces excessive or strategically selected muscle contraction and thereby improves dynamic rhytides. The mainstream literature has focused on efficacy, safety, injection anatomy, rheology, vascular complications, immunogenicity, and patient satisfaction [1,2,3,4,5,6]. However, aesthetic aging is also a mechanical phenomenon. Skin, subcutaneous fat, retaining ligaments, muscles, fascia, vessels, and lymphatics exist as a moving three-dimensional unit. Every facial expression, blink, smile, mastication cycle, sleep position, and postural change produces subtle tissue deformation. These repeated mechanical events are not merely external forces; they are biological signals interpreted by fibroblasts, endothelial cells, lymphatic endothelial cells, adipocytes, myocytes, immune cells, and keratinocytes.

The hypothesis explored in this review is that injectable aesthetic procedures may alter this dynamic mechanical environment. In an ideal procedure, this alteration is beneficial: filler restores structural support to an atrophic or deflated compartment, and botulinum toxin reduces pathologic or aesthetically undesirable hypercontraction. In less ideal situations, however, the injected material or chemically induced relaxation may reduce physiological mobility, compress local microvascular or lymphatic pathways, distort ECM tension, or diminish the normal expansion–contraction cycles that maintain mechanotransductive signaling. This review uses the term mechanical silence to describe a local tissue state in which dynamic motion, fluid exchange, and cell–matrix deformation are reduced or replaced by persistent static pressure or chemical immobilization.

The concept must be framed carefully. It would be inaccurate to claim that fillers or botulinum toxin necessarily block circulation, stop lymphatic drainage, or accelerate aging in all patients. Hyaluronic acid filler has been shown to stimulate collagen production in photodamaged skin, likely through mechanical stretching and fibroblast activation [7,8]. Botulinum toxin has also been investigated for effects on dermal fibroblasts, scar modulation, sebum, vascular tone, and wound healing, and not all data suggest vascular inhibition [9,10,11]. Therefore, the central question is not whether injectable procedures are intrinsically harmful, but whether certain patterns of use—excessive volume, repeated layering, poorly selected anatomic planes, periorbital overcorrection, or excessive muscle immobilization—may impair the dynamic mechanical environment required for optimal dermal homeostasis.

## 2. Methods and Scope of the Review

This article was designed as a structured narrative review and mechanistic hypothesis paper rather than a systematic review or quantitative meta-analysis. A literature search was conducted in PubMed/MEDLINE from database inception through 23 September 2026, supplemented by backward citation screening of relevant reviews, clinical studies, mechanistic studies, and consensus or guideline documents. Search concepts were combined using terms related to dermal filler, hyaluronic acid, calcium hydroxylapatite, poly-L-lactic acid, botulinum toxin, extracellular matrix, mechanotransduction, viscoelasticity, fibroblast, YAP/TAZ, tissue stiffness, cyclic strain, lymphatic drainage, lymphatic pumping, edema, vascular perfusion, ultrasound, elastography, cutometry, indocyanine green lymphography, pressure injury, mechanical loading, and tissue deformation.

Priority was given to human clinical studies when available. Experimental, in vitro, animal, biomaterials, and pressure-injury studies were included when they addressed a mechanistic question that could not be evaluated from direct aesthetic clinical evidence. Reviews and guidelines were used to contextualize established clinical complications, current imaging approaches, and the broader biological literature. Publications were selected for relevance to injectable-related tissue mechanics, ECM remodeling, fluid dynamics, muscle activity, imaging, or measurable mechanobiological endpoints. The literature unrelated to the mechanistic questions of this review was not used to support clinical causal claims.

For interpretation, the evidence was separated into three levels. The first level comprises established clinical observations, including filler-associated vascular complications, persistent edema, nodules and inflammatory reactions, as well as recognized botulinum-toxin effects such as paresis and muscle atrophy [1,2,3,4,5,6,12,13,14,15,16,17,18]. The second level comprises mechanistic evidence showing that fibroblasts respond to matrix stiffness, stretch and cyclic loading; that lymph transport depends on intrinsic contractility and extrinsic mechanical forces; and that sustained tissue deformation can alter perfusion and tissue viability [19,20,21,22,23,24,25,26,27,28,29,30,31,32,33,34,35,36,37,38,39,40,41,42,43,44,45,46,47,48,49]. The third level comprises the mechanical-silence hypothesis itself: the proposal that selected patterns of excessive or persistent filler-related loading or marked chemodenervation could alter local deformation, fluid exchange, or mechanotransductive signaling. This third level has not been established as a clinical adverse effect and requires prospective testing with objective mechanical, imaging, and physiological endpoints. Table 1 summarizes this evidence hierarchy.

## 3. Dermal Mechanobiology: Why Movement Matters

The dermis is a mechanosensitive organ. Fibroblasts attach to collagen, fibronectin, elastin-associated microfibrils, and other ECM molecules through integrins and focal adhesion complexes. Mechanical signals are transmitted through the cytoskeleton to the nucleus, where they influence gene expression, collagen production, matrix metalloproteinase activity, myofibroblast differentiation, and inflammatory signaling [19,20,21,22,23]. The YAP/TAZ pathway is a central mechanotransductive regulator, responding to tissue stiffness, cell shape, and cytoskeletal tension [25]. Substrate stiffness can direct stem-cell lineage specification, and by extension, dermal stiffness can influence fibroblast phenotype, myofibroblast activation, and ECM production [21,26].

In young and healthy skin, fibroblasts are elongated and mechanically engaged with an organized collagen network. In aged or photoaged skin, collagen fragmentation reduces mechanical tension, leading to fibroblast collapse, reduced procollagen synthesis, increased matrix metalloproteinase activity, and a self-perpetuating cycle of matrix deterioration [31,32,33,34,35]. This observation is critical for aesthetic medicine: dermal quality depends not only on adding material or reducing movement but also on restoring appropriate mechanical information to cells. Too little mechanical tension may lead to cellular inactivity; excessive or abnormal static stress may promote inflammation, fibrosis, or maladaptive remodeling.

The mechanical environment of the dermis is not purely elastic. Skin behaves as a heterogeneous, hydrated viscoelastic tissue in which the collagen–elastin network provides a major solid structural framework, while proteoglycans, glycosaminoglycans, bound water, and mobile interstitial fluid contribute to time-dependent deformation and load redistribution. Consequently, mechanical loading produces not only instantaneous deformation but also creep, stress relaxation, and fluid redistribution over time. Proteoglycan-rich ground substance is particularly relevant because its interaction with water contributes to tissue hydration, matrix spacing, and transmission of mechanical loads between the fibrillar ECM and the interstitial fluid phase. From this perspective, an injectable material may change not only static stiffness but also local relaxation behavior, tissue recoil, interstitial pressure, and fluid movement. More broadly, viscoelastic biomaterial studies demonstrate that cellular mechanosensing can depend on the relationship between matrix relaxation timescale and cell-adhesion dynamics [50]. However, these experimental observations do not establish a specific viscoelastic threshold for human facial skin after aesthetic injection and should be regarded as mechanistic context rather than direct clinical evidence.

**Figure 1:**
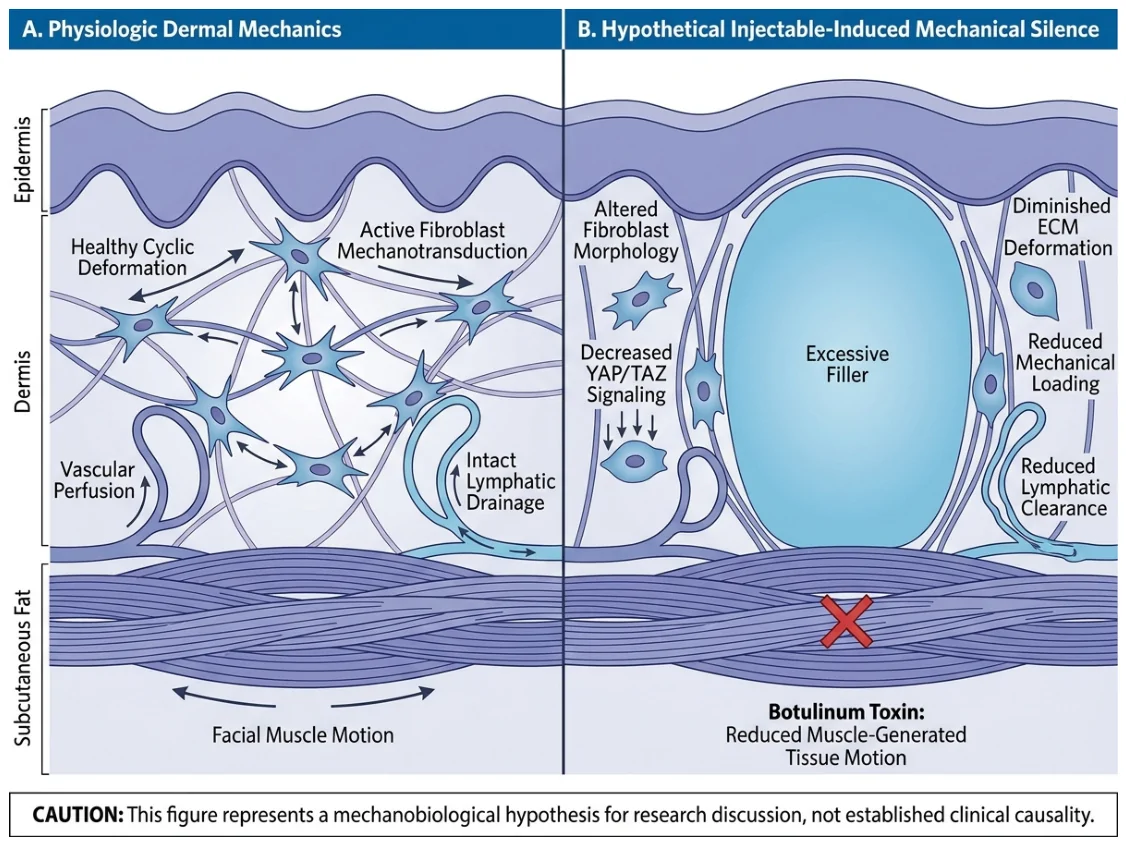
Dynamic dermal mechanotransduction versus the proposed injectable-related mechanical-silence framework. The left panel depicts physiological soft-tissue motion, ECM deformation, perfusion, and lymphatic clearance as components of a dynamic mechanical environment. The right panel illustrates hypothetical changes that could occur under selected conditions of excessive filler-related loading or marked reduction in muscle-generated tissue motion. The pathways shown are conceptual and hypothesis-generating; they do not represent established causal pathways or demonstrated adverse effects of dermal fillers or botulinum toxin.

The dermis is also exposed to cyclic deformation. Facial expression, blinking, smiling, chewing, talking, and head movement produce repeated low-amplitude expansion and recoil. These cycles may help maintain fluid exchange and mechanotransductive tone. The analogy is not perfect, but it is conceptually similar to other tissues in which cyclic loading maintains tissue health, whereas prolonged static loading produces injury. In fibroblast culture, cyclic stretch and mechanical constraints can reorganize cells, affect integrin distribution, and alter ECM gene expression [23,24,28,29]. Therefore, the skin should be understood as a dynamic mechanochemical system rather than a static canvas for filling and paralysis. The distinction between physiological dynamic loading and the proposed mechanical-silence state is illustrated schematically in Figure 1.

## 4. Dermal Fillers: Beneficial Support Versus Chronic Mass Effect

Hyaluronic acid fillers are not biologically inert. Clinical and histological studies indicate that cross-linked hyaluronic acid can integrate into dermal tissue, alter local mechanics, stimulate fibroblast elongation, and induce new collagen production [7,8,51,52]. This effect is often beneficial because photodamaged skin contains fragmented collagen and collapsed fibroblasts. By restoring mechanical stretch, a well-placed filler can re-engage fibroblasts and partially restore a youthful matrix environment [7,8,31,32,33,34,35]. Thus, the proposed mechanical-silence hypothesis should not be confused with a blanket anti-filler argument.

The concern arises when filler becomes excessive, persistent, poorly distributed, or positioned in a region with limited drainage capacity. Filler has rheological properties such as elasticity, cohesivity, swelling behavior, viscosity, and resistance to deformation. These properties determine how a product spreads, integrates, displaces tissue, resists compression, and interacts with the surrounding ECM. A small amount of filler in the correct plane may restore support, whereas repeated injections in a superficial or lymphatic-rich region may create a persistent mass that changes pressure gradients and tissue mobility. In the periorbital and malar regions, prolonged edema after hyaluronic acid injection has been reported, and hyaluronidase may improve selected cases [12,13,53]. The mechanism is likely multifactorial: hydrophilic water retention, inflammatory susceptibility, venous stasis, local lymphatic compromise, injection volume, anatomical predisposition, and prior surgery or aging-related tissue laxity.

Importantly, filler classes should not be treated as mechanically or biologically equivalent. Cross-linked HA products are hydrated viscoelastic gels that provide immediate volume and load sharing, with product-specific differences in elasticity, viscosity, cohesivity, swelling behavior, and tissue integration [7,8,51,52]. Calcium hydroxylapatite (CaHA) consists of CaHA microspheres suspended in a carrier gel and combines an early structural effect with subsequent tissue remodeling and collagen-related biostimulation; its particulate scaffold and degradation behavior differ substantially from those of HA [54]. Poly-L-lactic acid (PLLA), in contrast, is a biodegradable particulate biostimulator whose clinical effect develops progressively through host-cell responses and collagen remodeling rather than through persistence of a hydrated gel-like volume [55]. Therefore, hypotheses concerning hydrophilic swelling, static gel loading, interstitial-fluid redistribution, or reversibility with hyaluronidase are most directly applicable to HA and should not be generalized without qualification to CaHA or PLLA. The major mechanobiological distinctions among these filler classes are summarized in Table 2.

From a mechanobiological perspective, excessive filler may influence three linked processes. First, it may change the deformation pattern of the ECM. Instead of allowing the tissue to expand and recoil freely, a bolus or sheet of filler may impose constant stretch, compression, or stiffness. Second, it may alter interstitial fluid movement. Lymphatic capillaries are low-pressure structures that can be affected by tissue deformation and pressure gradients; lymphatic dysfunction has been observed in compressed skin models [43]. Third, filler may modify vascular flow. In the most serious setting, intravascular or perivascular filler can cause vascular occlusion, skin necrosis, or blindness [3,4,5,6]. In less severe settings, persistent pressure or edema may contribute to microcirculatory inefficiency, although this requires direct measurement.

**Figure 2:**
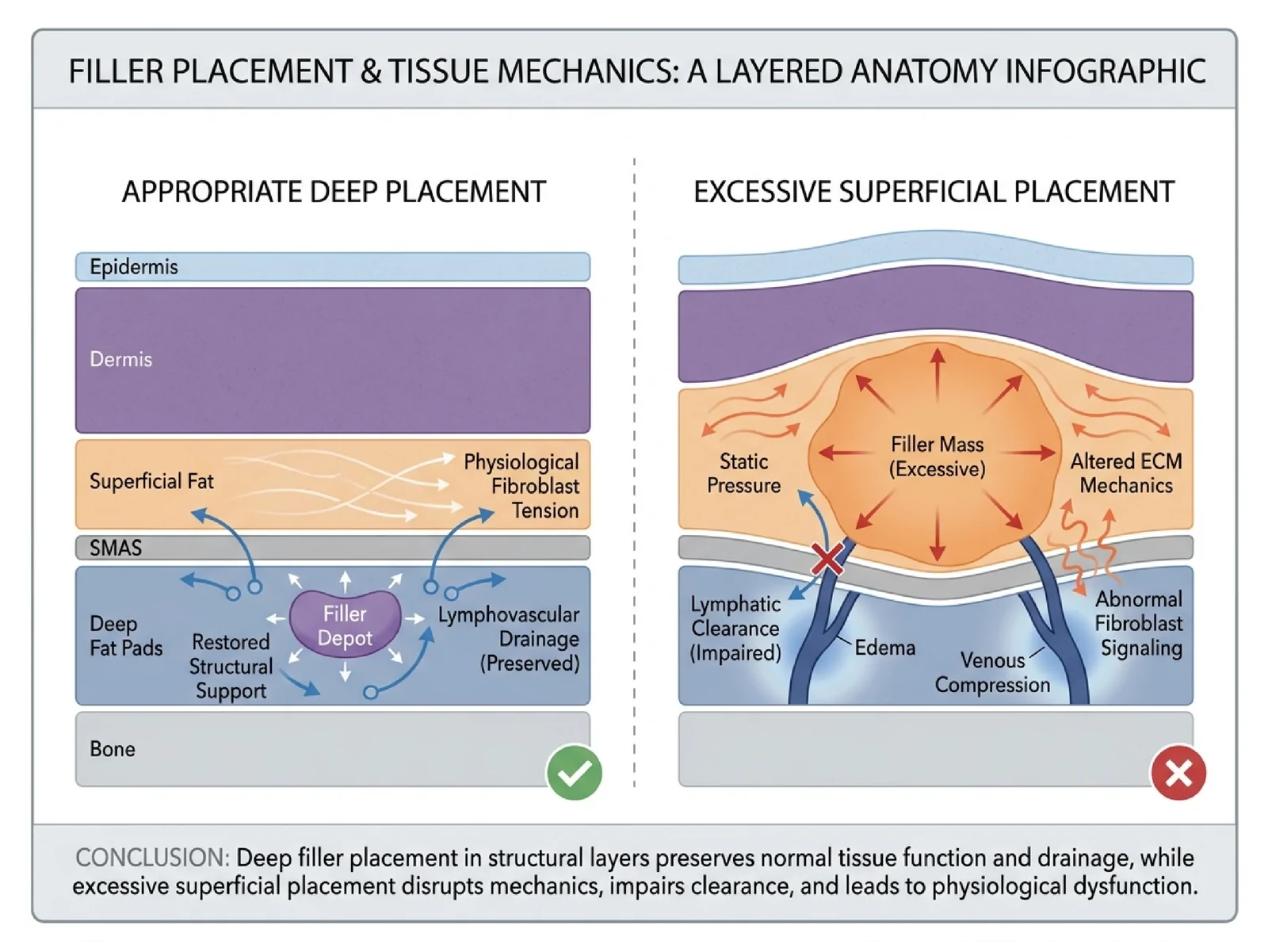
Conceptual model of filler placement, ECM deformation, and lymphovascular context. The figure contrasts appropriately integrated structural support with a hypothetical scenario in which excessive or superficially distributed filler could be associated with altered local stiffness, swelling, ECM strain, or fluid clearance. HA fillers can also promote fibroblast activation and collagen remodeling through mechanical stretching when appropriately used. The pathways illustrated here are conceptual and should not be interpreted as established causal consequences of filler treatment.

It is useful to distinguish regenerative mechanical stretch from maladaptive static deformation. Wang and colleagues showed that cross-linked hyaluronic acid in photodamaged skin stimulated collagen synthesis and produced stretched, biosynthetically active fibroblasts [7]. Turlier and colleagues similarly associated collagen production with mechanical stretching of the dermal ECM after cross-linked hyaluronic acid filler [8]. These studies support the idea that mechanical stretch can be beneficial. However, the beneficial signal may depend on an appropriate dose, plane, stiffness, and integration pattern. Overcorrection may shift the tissue from physiological mechanotransduction to persistent mechanical distortion. These contrasting mechanical scenarios are illustrated conceptually in Figure 2.

## 5. Botulinum Toxin: Dynamic Wrinkle Control and the Question of Mechanical Quiescence

Botulinum toxin type A acts primarily by blocking acetylcholine release at the neuromuscular junction through cleavage of SNAP-25 and disruption of SNARE-dependent vesicle fusion [14,15,56,57]. In aesthetic medicine, this produces temporary reduction in muscle contraction and improvement of dynamic wrinkles. The desired effect is movement modulation, not tissue destruction. In clinical practice, precise dosing can reduce excessive contraction while preserving natural expression.

The mechanobiological question is different. If repeated or high-dose chemodenervation substantially reduces muscle activity, the overlying skin and subcutaneous tissue may receive less cyclic movement. Facial muscles are not merely wrinkle-generating structures; they also create local tissue deformation, contribute to venous and lymphatic movement, and transmit mechanical information to the dermis through fascial, ligamentous, and cutaneous insertions. Reduction in contraction may therefore reduce both unwanted wrinkle-forming compression and potentially useful low-amplitude mechanical stimulation.

Evidence from the skeletal muscle literature shows that botulinum toxin-induced paralysis can produce muscle atrophy and molecular changes related to denervation [18,58]. More recent studies have explored differences between botulinum toxin paralysis and neurectomy in fibrosis, NF-kB, and TGF-beta signaling [59]. These data should not be directly translated to cosmetic facial doses without caution, but they demonstrate that chemical reduction in movement is biologically active. In the dermis, botulinum toxin has been reported to influence fibroblast collagen production and degradation in vitro [9], while other studies suggest possible effects on scar biology and wound healing. These findings are complex: botulinum toxin may reduce mechanical tension and scar formation in some settings, but reduced mechanical activity may also contribute to tissue quiescence if excessive or prolonged.

The vascular and lymphatic interpretation requires particular caution. A proposed mechanism is that marked reduction in facial muscle excursion could reduce one component of extrinsic tissue movement that contributes to regional fluid redistribution; however, direct evidence that aesthetic botulinum toxin treatment produces clinically meaningful impairment of facial lymphatic pumping is currently lacking. Experimental data indicate that lymphatic endothelial cells are largely unaffected by botulinum toxin A under the conditions studied, apart from limited changes in cord-like structure formation [10]. Human vascular observations have also reported increased rather than reduced skin perfusion and vessel diameter in selected settings [11]. Recent reviews of facial botulinum toxin complications continue to identify predominantly neuromuscular and functional adverse effects rather than a demonstrated chronic lymphatic syndrome [60]. Accordingly, reduced lymphatic pumping, long-term dermal mechanical quiescence, and persistent alterations in fluid clearance should remain explicitly labeled as hypotheses requiring prospective human measurement.

## 6. Lymphatic Drainage, Vascular Circulation, and Facial Motion

The lymphatic system maintains interstitial-fluid balance, immune surveillance, lipid transport, and macromolecule clearance. Unlike the arterial circulation, lymphatic flow depends on intrinsic lymphatic contractility, pressure gradients, valves, respiration, arterial pulsation, and extrinsic tissue movement [37,38,39,40,41,42]. In the face, thin tissues, repeated blinking, facial muscle contraction, and gravity-dependent postural changes likely contribute to fluid redistribution. The periorbital and malar regions are particularly susceptible to visible fluid accumulation because of thin skin, ligamentous boundaries, age-related tissue laxity, venous anatomy, and lymphatic complexity.

Fillers and botulinum toxin could influence this system through different mechanisms. Fillers may create a physical volume, hydrophilic reservoir, or local stiffness that changes interstitial-fluid pressure and lymphatic clearance. Botulinum toxin may reduce the extrinsic muscle-motion component of lymphatic flow. Both may interact with venous drainage: filler may contribute to venous stasis or local pressure in certain planes, while botulinum toxin may either reduce vasoconstrictive tone or increase perfusion in selected settings [11]. Therefore, the direction of effect is not uniform.

**Figure 3:**
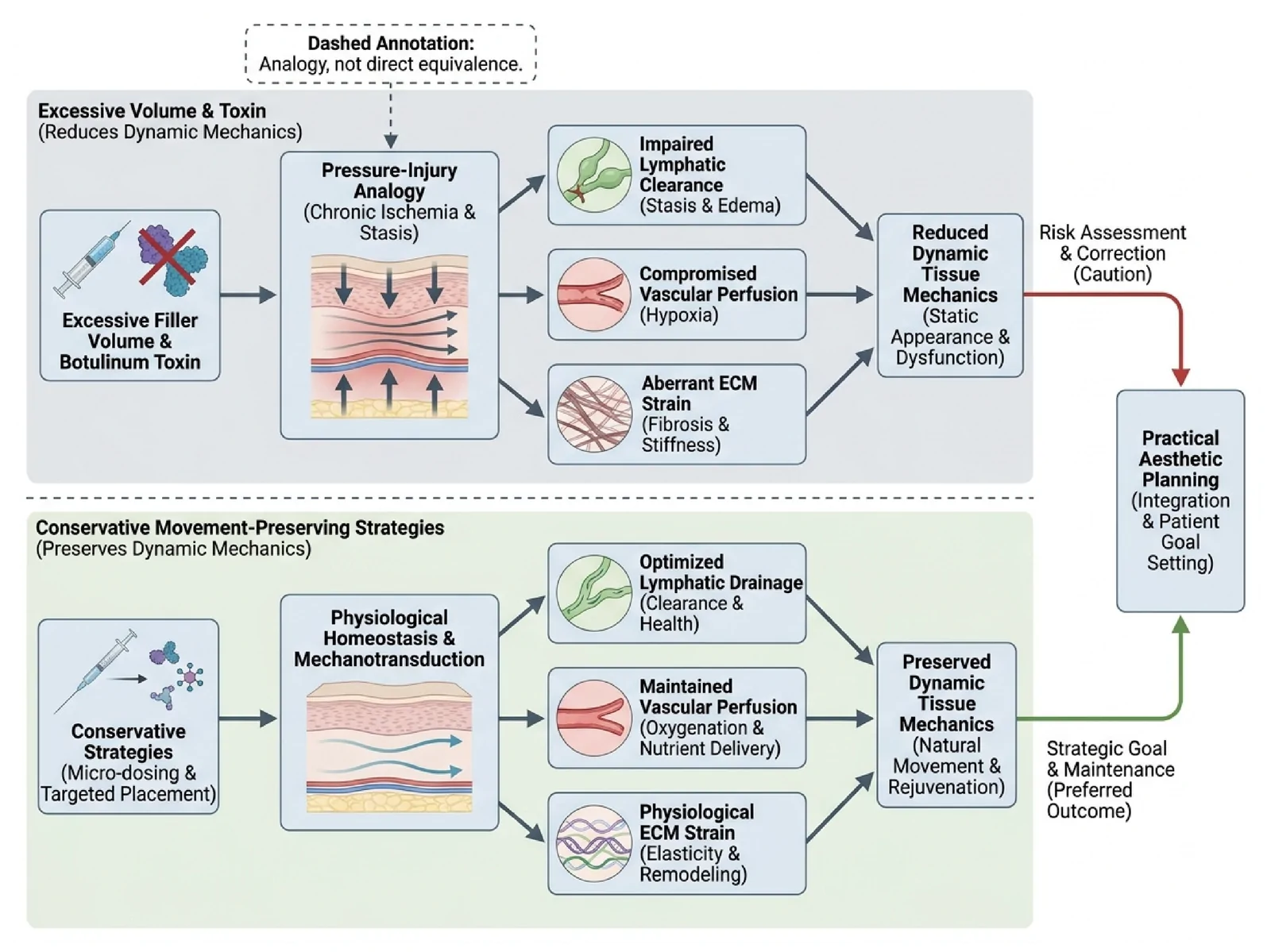
Pressure-injury biology as a limited conceptual analogy for injectable-related mechanical hypotheses. The diagram uses pressure-injury research only to illustrate the general distinction between prolonged static loading and dynamic tissue deformation. Aesthetic filler treatment and botulinum toxin treatment differ fundamentally from pressure injury in loading magnitude, duration, anatomy, perfusion effects, and clinical context. The pathways shown are therefore conceptual and should not be interpreted as established causal pathways of injectable treatment.

Pressure-injury biology provides only a limited conceptual analogy. Sustained tissue deformation can impair perfusion, fluid exchange, and tissue viability, and repositioning is an established component of pressure-injury prevention [44,46,47,48]. Experimental mechanical stimulation, including vibration, has also been investigated in pressure-injury models [49]. However, the magnitude, duration, tissue geometry, vascular consequences, and clinical context of pressure injury are fundamentally different from those of aesthetic filler placement or botulinum toxin treatment. Accordingly, this literature is used here only to illustrate the general biological principle that the duration and pattern of mechanical loading can influence soft-tissue physiology; it does not provide evidence that injectable procedures reproduce pressure-injury mechanisms. The boundaries of this analogy are summarized in Figure 3.

## 7. Molecular Pathways Potentially Affected by Reduced ECM Expansion and Recoil

The phrase ECM expansion and contraction refers to the repeated deformation of collagen, elastin, ground substance, and interstitial-fluid spaces during normal tissue motion. At the cellular level, these events are sensed through integrins, focal adhesion kinase, talin, vinculin, stretch-activated ion channels, the actin cytoskeleton, nuclear lamins, and transcriptional regulators such as YAP and TAZ [19,20,21,22,23,24,25,26,27]. Mechanical input also interacts with growth-factor signaling. TGF-beta can be stored in a latent ECM-bound form, and mechanical tension can affect its activation; myofibroblast differentiation is similarly linked to matrix stiffness, actomyosin tension, and alpha-smooth-muscle-actin expression [28,29]. Therefore, the dermis receives biochemical signals partly because it is mechanically deformed.

A filler placed in the dermis or subcutaneous tissue can alter this signaling in two opposite directions. When it restores lost tension to fragmented collagen, it may improve fibroblast spreading and matrix synthesis [7,8]. When it produces focal stiffness, chronic swelling, or a poorly mobile gel-tissue interface, it may reduce the normal pattern of cyclic strain. In that setting, fibroblasts may receive a constant static signal instead of physiologic oscillatory input. The resulting phenotype could be quiescent, fibrotic, inflammatory, or matrix-degrading depending on local chemistry, stiffness, oxygenation, and immune response. This is why the same general product category can be associated with both regenerative collagen stimulation and undesirable stiffness or edema in different patients.

Botulinum toxin affects the same system from the opposite direction. It does not add a physical mass, but it reduces the movement generator. In the upper face, glabellar complex, orbicularis oculi, frontalis, mentalis, depressor anguli oris, and platysma, muscle contraction repeatedly changes dermal strain. Controlled reduction in hypercontraction reduces repeated folding, which is beneficial. Excessive reduction may lower tissue deformation below a biologically useful range. This is similar to the difference between correcting harmful overuse and inducing disuse. Disuse biology is well recognized in skeletal muscle, where loss of activity is linked to atrophy, altered gene expression, and changes in connective tissue [18,58,59]. The facial dermis is not skeletal muscle, but it is mechanically coupled to muscle.

This section proposes a hypothetical U-shaped mechanobiological framework for future investigation. Repetitive excessive compression contributes to dynamic wrinkle formation, whereas markedly reduced movement may theoretically reduce cyclic mechanical input. Whether this reduction reaches a biologically meaningful threshold in human facial tissues has not been established. Accordingly, the proposed intermediate zone—combining reduction in undesirable folding with structural support and preservation of physiological motion—should be regarded as a testable mechanobiological concept rather than a validated treatment target.

## 8. Clinical Phenotypes That Could Be Reinterpreted Through the Mechanical-Silence Framework

Several clinical observations can be used to generate, but not confirm, the mechanical-silence hypothesis. Persistent malar or periorbital edema after HA filler is an established clinical phenomenon, with proposed contributors including product hydrophilicity, injection plane, anatomy, inflammation, venous factors, and lymphatic susceptibility [12,13,53,61]. Whether altered tissue deformation or reduced muscle-generated motion independently contributes to this edema has not been demonstrated. Likewise, cumulative filler burden may be associated clinically with palpable firmness, overcorrection, altered contour, or reduced apparent mobility, but a direct causal relationship between cumulative filler exposure and abnormal mechanotransductive signaling has not been established.

Other observations—including prolonged swelling after subsequent procedures or a subjective mismatch between wrinkle reduction and natural facial animation—are even less specific and may have multiple alternative explanations. These phenomena should therefore not be regarded as manifestations of a defined mechanical-silence syndrome. Their relevance is primarily hypothesis-generating: they identify patient groups in whom objective measurements of filler burden, tissue stiffness, dynamic deformation, perfusion, and regional fluid clearance could be particularly informative.

## 9. Proposed Concept: Injectable Procedure-Related Mechanical Silence

For the purposes of this review, injectable procedure-related mechanical silence is defined as a hypothetical local state characterized by a measurable reduction or distortion of normal dynamic tissue deformation, rather than as a recognized diagnosis or established complication. Potential contributing variables include persistent local stiffness or mass effect, reduced expansion–recoil cycles, altered interstitial-fluid movement, or marked reduction in muscle-driven tissue excursion. The proposed construct therefore requires operational measurement before it can be assigned clinical significance.

Candidate mechanical endpoints include dynamic strain amplitude during standardized facial movement, deformation and recoil measured over repeated loading cycles, regional stiffness measured by elastography, superficial skin distensibility and elastic recovery, muscle excursion, and the relationship between filler burden and movement of adjacent soft-tissue layers. Candidate physiological endpoints include lymphatic transit, regional perfusion, edema volume, and interstitial-fluid-related imaging findings. Molecular or translational studies could additionally evaluate focal-adhesion signaling, YAP/TAZ localization, cytoskeletal organization, and ECM-remodeling markers. Importantly, no validated facial-skin strain threshold currently defines YAP/TAZ inactivation or “mechanical silence” after aesthetic injectable treatment. Dynamic strain amplitude and related measures should therefore be treated as continuous experimental variables rather than clinical cutoffs.

Within this framework, HA-dominant, chemodenervation-dominant, and combined exposure patterns can be investigated separately because they represent different mechanical perturbations. HA may change local volume, hydration, stiffness, or deformation patterns, whereas botulinum toxin primarily changes the active movement generator. CaHA and PLLA require separate interpretation because their particulate and biostimulatory behavior differs from that of HA. These relationships remain hypotheses until longitudinal human studies demonstrate reproducible associations between injectable exposure and objective mechanical or physiological endpoints. The framework is therefore intended to generate testable research questions, not to define a new adverse-event diagnosis or treatment indication.

## 10. Clinical Boundaries and Research Priorities

The mechanical-silence hypothesis should not be used to create a separate clinical treatment protocol. Current aesthetic practice should continue to rely on established principles of patient selection, anatomy, product characteristics, appropriate injection plane and volume, recognized complication management, and individualized botulinum toxin dosing. In patients with persistent swelling or suspected residual HA filler, high-frequency ultrasound can provide structural information regarding filler location and adjacent tissue changes, while hyaluronidase may be considered when clinically indicated for HA-related filler burden [12,13,53]. These practices are supported independently of the mechanical-silence hypothesis.

Manual lymphatic drainage, facial exercise, vibration, microcurrent, transcutaneous electrical stimulation, therapeutic ultrasound, and radiofrequency should not currently be presented as evidence-based interventions for preventing or treating injectable-related mechanical silence. Direct clinical evidence demonstrating that these modalities normalize injectable-associated mechanobiology is insufficient. Their relevance in this review is therefore limited to future research: controlled studies could determine whether any modality measurably changes edema, lymphatic transit, tissue stiffness, deformation, or recovery after specific injectable procedures. Until such evidence exists, these approaches should not be inferred from the present hypothesis as routine post-injection recommendations.

Future studies should assess both appearance and physiology. Candidate outcomes include filler burden and location, regional stiffness, dynamic tissue strain, elastic recoil, muscle excursion, dermal or subcutaneous thickness, lymphatic transit, vascular perfusion, edema volume, and patient-reported stiffness or altered mobility. Objective endpoints should be measured longitudinally and interpreted according to filler class, product properties, dose, injection plane, anatomical region, concomitant botulinum toxin exposure, and baseline tissue characteristics.

## 11. Proposed Research Designs

Several research designs could test the mechanical-silence hypothesis. A first study could be a cross-sectional ultrasound and elastography study comparing patients with no filler history, moderate filler history, and extensive repeated filler history. Outcomes would include filler burden, tissue stiffness, dynamic deformation during expression, dermal echogenicity, and malar edema score. A second study could examine periorbital lymphatic transit using indocyanine green lymphography or near-infrared imaging before and after tear trough or midface filler injection. A third study could evaluate botulinum toxin effects on periorbital fluid dynamics by measuring eyelid blink amplitude, orbicularis oculi excursion, lymphatic imaging, and skin edema over time.

A fourth study could evaluate the combined injectable phenotype. Patients undergoing filler plus botulinum toxin could be compared with patients undergoing filler alone, botulinum toxin alone, and energy-based procedures that preserve movement. The endpoint would not be whether one treatment is “good” or “bad,” but whether different procedures produce measurable differences in tissue mechanics and drainage. A fifth translational study could use ex vivo skin or three-dimensional fibroblast collagen gels to compare cyclic stretch, static compression, filler contact, and botulinum-toxin-conditioned media on COL1A1, COL3A1, ELN, MMP1, TGFB1, ACTA2, YAP/TAZ nuclear localization, inflammatory cytokines, and lymphangiogenic markers.

The most clinically useful trial would be a prospective, anatomy-stratified study of patients receiving conservative versus high-volume filler strategies. Patients would be followed with ultrasound, three-dimensional imaging, edema scoring, and elastography for 12 to 24 months. The study could determine whether cumulative filler burden predicts tissue stiffness, delayed edema resolution, or reduced dynamic deformation. A similar prospective botulinum toxin study could determine whether repeated high-dose immobilization produces measurable changes in muscle thickness, local perfusion, lymphatic clearance, and dermal quality compared with low-dose movement-preserving protocols.

## 12. Regional Considerations: Periorbital, Midface, Perioral, and Neck

The mechanical-silence hypothesis is most relevant in regions where thin soft tissue, complex lymphovascular anatomy, repeated motion, and aesthetic overcorrection intersect. The periorbital region is the clearest example. The orbicularis oculi produces frequent low-amplitude blinking, which mechanically deforms eyelid and malar soft tissue. Tear trough and midface filler may improve hollowing, but this region is also prone to edema. When filler volume, hydrophilicity, superficial placement, or pre-existing malar fluid tendency is combined with reduced orbicularis activity, the drainage environment may become less dynamic. This does not mean that periorbital filler or toxin should be avoided universally. It instead identifies the periorbital region as a priority setting for prospective study because filler properties, local anatomy, edema susceptibility, and orbicularis motion coexist within a mechanically complex environment. Whether different degrees of hollow correction or muscle-motion reduction produce measurable differences in lymphatic or tissue-mechanical endpoints remains to be established.

In the midface, filler is often used to restore structural support and lifting vectors. Appropriate deep placement can improve cheek projection and reduce nasolabial folding. Yet repeated injections into superficial compartments may limit glide between layers and may increase the visible stiffness of smiling or animation. The midface is not a static shelf; it deforms with smiling, chewing, and eye closure. Therefore, product selection and plane should be matched not only to the desired projection but also to dynamic movement. Ultrasound may help differentiate residual filler from edema, fat lobules, scar tissue, or inflammatory nodules.

In the perioral region, botulinum toxin and filler have particularly complex mechanical consequences. Lip filler can improve contour and hydration, but excessive product may alter orbicularis oris mechanics, speech, drinking, smiling, and local tissue mobility; whether it independently impairs lymphatic clearance requires direct investigation. Botulinum toxin in perioral depressors or elevators may improve asymmetry and hyperactivity, but excessive weakening can change oral competence and the mechanical relationship between lip elevators and depressors. Because perioral tissues are highly mobile, preserving function is central to tissue health.

In the neck and lower face, treatments involving the platysma, depressor anguli oris, mentalis, masseter, salivary glands, or jawline fillers can alter soft-tissue movement and contour. Because the neck contains an extensive superficial lymphatic network and undergoes substantial motion during swallowing and head rotation, this region may be suitable for future studies examining whether injectable-related changes in motion are associated with measurable changes in fluid dynamics. At present, however, a direct effect on lymphatic direction or long-term lymphatic function has not been established. Jawline filler and platysmal toxin may be visually powerful but can also create static borders if overused. The neck has a rich superficial lymphatic network and significant movement during swallowing and head rotation. A movement-preserving strategy may therefore be preferable to an approach focused only on immobilizing bands and sharpening contours.

## 13. Practical Research Biomarkers and Imaging Endpoints

A major limitation of the current injectable literature is that treatment outcomes are predominantly documented by static photographs, aesthetic scales, satisfaction scores, or adverse-event reporting. Testing the mechanical-silence hypothesis requires complementary structural, mechanical, vascular, and lymphatic measurements. High-frequency ultrasound can characterize filler location, distribution, depth, edema, nodules, and adjacent vascular anatomy [62]. Suction-based cutometry can quantify surface deformation and elastic recovery, although acquisition and interpretation require rigorous standardization [63]. Indocyanine green lymphography can visualize superficial lymphatic pathways and transit but remains investigational for this specific facial injectable application [64]. Shear-wave elastography can quantify regional tissue stiffness and has previously been used to measure facial soft-tissue stiffness in aesthetic research [65]. The candidate tools, measurable parameters, potential mechanobiological relevance, and major interpretive limitations are summarized in Table 3.

Molecular endpoints could be collected with minimally invasive approaches. Tape stripping, suction blister fluid, or microneedle sampling may allow measurement of inflammatory cytokines, MMPs, oxidative-stress markers, and ECM-related proteins without large biopsies. Optional biopsy in selected research settings could evaluate COL1A1, COL3A1, ELN, MMP1, TGFB1, ACTA2, CD31, LYVE-1, podoplanin, PROX1, CD68, CD163, YAP/TAZ localization, and p16 or p21 senescence markers. For botulinum toxin studies, muscle ultrasound can measure thickness and contractile excursion before and after treatment. For filler studies, ultrasound can quantify cumulative filler burden and relationship to lymphatic-sensitive planes.

Dynamic tissue deformation may be a particularly informative endpoint because static appearance does not fully describe how skin, subcutaneous tissue, muscle, and filler move during expression. Standardized measurements during smiling, blinking, or other reproducible facial maneuvers could quantify regional strain, displacement, and recoil. Such measurements should initially be treated as research endpoints rather than as validated measures of “mechanical naturalness,” because normative ranges and clinically meaningful thresholds have not yet been established.

## 14. Limitations of the Hypothesis

The mechanical-silence concept has several important limitations. First, direct longitudinal human evidence is currently lacking; much of the proposed framework is extrapolated from established filler complications, general mechanotransduction, lymphatic physiology, biomaterials research, skeletal-muscle studies, and pressure-injury biology. Second, fillers are heterogeneous materials, and HA, CaHA, PLLA, and other products cannot be assumed to produce equivalent mechanical or biological effects. Third, botulinum toxin effects depend on formulation, dose, muscle, anatomical region, treatment interval, and baseline function, and direct evidence linking aesthetic chemodenervation to impaired facial lymphatic pumping is insufficient. Fourth, proposed mechanical endpoints such as strain amplitude, stiffness, recoil, and dynamic tissue displacement do not yet have validated diagnostic thresholds for this application. Fifth, apparently unfavorable mechanical interpretations must be balanced against evidence that HA can stimulate collagen remodeling and that botulinum toxin may increase skin perfusion in selected settings [7,8,11].

Accordingly, mechanical silence should remain a hypothesis-generating construct rather than a clinical diagnosis, adverse-event category, or basis for treatment recommendations. Its value will depend on whether prospective studies can demonstrate reproducible relationships among injectable exposure, objective tissue-mechanical measurements, fluid dynamics, molecular signaling, and clinically meaningful outcomes.

## 15. Conclusions

Dermal fillers and botulinum toxin alter facial soft tissues through different and, in many respects, beneficial mechanisms. HA fillers can restore structural support and stimulate collagen remodeling, whereas CaHA and PLLA have distinct material and biostimulatory profiles. Botulinum toxin reliably reduces selected muscle activity and dynamic rhytides. These established effects should be distinguished from the hypothesis proposed in this review.

Injectable procedure-related mechanical silence is proposed as a research framework for asking whether selected patterns of altered tissue loading or markedly reduced tissue motion are associated with measurable changes in dynamic deformation, stiffness, fluid movement, perfusion, or cell–matrix signaling. Current evidence does not establish mechanical silence as a clinical syndrome, demonstrate a causal relationship between aesthetic injectable treatment and chronic lymphatic dysfunction, or support specific adjunctive therapies for its prevention or treatment. Pressure-injury biology provides only a limited conceptual comparison and should not be interpreted as evidence of equivalent tissue loading or pathology.

The principal next step is therefore measurement rather than stronger clinical inference. Prospective, anatomy-stratified studies integrating ultrasound, elastography, cutometry, lymphatic imaging, perfusion assessment, standardized facial-motion analysis, and molecular endpoints are needed to determine whether the proposed mechanobiological relationships occur in humans and whether they have clinically meaningful consequences.

## Figures and Tables

**Table 1:** Evidence map for the mechanical-silence hypothesis.

Domain	Established Evidence	Mechanistic Extension	Clinical Caution
**Filler vascular risk**	Rare vascular occlusion, necrosis, blindness, and ischemic events are described.	Perivascular pressure or persistent edema may influence microcirculation, but direct proof is limited.	Avoid intravascular injection; use established anatomy- and safety-based injection principles. Ultrasound may assist selected cases.
**Filler lymphatic/edema risk**	Periorbital and malar edema after HA filler is reported, particularly in susceptible anatomical regions.	Hydrophilicity, injected volume, plane, local stiffness, venous factors, and lymphatic susceptibility may interact; the independent contribution of altered tissue mechanics remains unproven.	Assess pre-existing edema and filler burden when clinically relevant; ultrasound may assist evaluation, and hyaluronidase may be considered for confirmed or strongly suspected HA-related filler burden when otherwise clinically indicated.
**BoNT movement effect**	BoNT blocks acetylcholine release and reduces muscle contraction; paresis and muscle atrophy are recognized biological effects.	Reduced facial movement could alter cyclic mechanical input and extrinsic tissue motion, but a clinically meaningful chronic reduction in facial lymphatic pumping has not been established.	Use established indication-, anatomy-, product-, and patient-specific dosing principles. Whether differing degrees of movement preservation influence long-term tissue mechanics or lymphatic function remains a research question.
**BoNT vascular effect**	Available human observations do not support universal vascular blockage; increased perfusion or vessel diameter has been reported in selected settings.	Neurovascular responses may vary by region, dose, depth, and clinical context.	Do not infer generalized vascular impairment from the mechanical-silence hypothesis; use objective perfusion endpoints in future studies.
**Pressure-injury analogy**	Sustained loading and immobility can damage tissue in pressure-injury settings; repositioning is an established preventive strategy.	This literature illustrates that loading pattern and duration can influence soft-tissue physiology, but aesthetic injectables differ fundamentally in magnitude, duration, anatomy, and clinical context.	Use the analogy only as a conceptual model and not as evidence of direct equivalence or causality.

**Table 2:** Comparative mechanobiological characteristics of major injectable filler classes.

Feature	Hyaluronic Acid (HA)	Calcium Hydroxylapatite (CaHA)	Poly-L-Lactic Acid (PLLA)
**Material architecture**	Cross-linked hydrophilic polysaccharide gel; behavior varies with concentration, cross-linking, particle/gel architecture, cohesivity, and rheology.	CaHA microspheres suspended in a carboxymethylcellulose-based carrier gel; provides a particulate scaffold plus carrier-mediated initial correction.	Biodegradable PLLA microparticles supplied for reconstitution; a particulate biostimulator with limited immediate structural effect from the carrier/reconstitution volume.
**Primary early mechanical effect**	Immediate space filling, hydration, tissue displacement/support, and viscoelastic load sharing; magnitude depends strongly on product rheology and injection plane.	Immediate structural support from the carrier/particle suspension, followed by tissue-driven remodeling as the carrier dissipates.	Less dependent on persistent gel volume; early mechanical effects are modest and treatment effects develop progressively through tissue remodeling.
**Water interaction**	Strong water-binding behavior; hydrophilicity can influence swelling and interstitial-fluid balance, with substantial product-to-product variability.	Not primarily a hydrophilic gel filler; water-related swelling is less central to its mechanism than for HA.	Not primarily hydrophilic; clinical effects are not driven by water-binding gel expansion.
**Cell–matrix interaction**	Can mechanically stretch surrounding ECM and fibroblasts; human and experimental evidence supports fibroblast activation and collagen synthesis after selected cross-linked HA fillers.	Microspheres act as a biostimulatory scaffold and are associated with fibroblast activity, neocollagenesis, and ECM remodeling.	Microparticles provoke a controlled host response involving macrophage–fibroblast signaling and progressive collagen deposition.
**Degradation/persistence**	Gradual enzymatic and hydrolytic degradation; HA can be degraded with hyaluronidase, although clinical reversibility depends on product, location, amount, and timing.	Carrier gel is resorbed; CaHA particles are progressively degraded/resorbed while collagenous remodeling can persist beyond the initial carrier phase.	PLLA undergoes hydrolytic degradation over time; biostimulatory effects develop gradually and newly formed collagen can persist after substantial polymer degradation.
**Mechanobiological implication for this review**	Most directly relevant to hypotheses involving local stiffness, static volume, hydrophilic swelling, altered deformation, and interstitial-fluid redistribution.	More relevant to interactions between particulate scaffold mechanics and subsequent collagen remodeling; mass effect should not be assumed to behave identically to HA.	More relevant to delayed tissue-mediated remodeling and collagen accumulation than to persistent gel-like static loading; effects should be framed separately from HA.
**Evidence boundary**	Material/rheological behavior and collagen stimulation are supported, but long-term effects on lymphatic pumping or a defined mechanical-silence state have not been established clinically.	Biostimulation and ECM remodeling are supported, but direct human evidence linking CaHA to mechanical-silence metrics is lacking.	Biostimulation and collagen remodeling are supported, but direct human evidence linking PLLA to mechanical-silence metrics is lacking.

**Table 3:** Candidate objective tools and endpoints for testing injectable-related changes in tissue mechanics and fluid dynamics.

Tool	Primary Measurable Parameters	Potential Relevance to the Hypothesis	Key Limitations/Interpretation
**Shear-wave elastography (SWE)**	Shear-wave velocity and derived regional tissue stiffness/elastic modulus; repeat measurements during standardized conditions may characterize spatial stiffness differences.	Can test whether treated regions or areas with different filler burden show measurable differences in tissue stiffness and whether stiffness changes over time.	Stiffness is influenced by anatomy, probe pressure, acquisition technique, device settings, tissue anisotropy, edema, and filler type. No validated facial mechanical-silence cutoff exists.
**Cutometry/suction-based skin testing**	Suction-induced deformation, distensibility, immediate and delayed recoil, and derived elasticity/viscoelasticity indices.	Can quantify superficial skin deformation and recovery and may complement deeper imaging when testing changes in dynamic mechanical behavior.	Primarily assesses superficial tissue; results depend on probe geometry, suction protocol, site, hydration, temperature, and device-specific parameters. Standardization is essential.
**Indocyanine green (ICG) lymphography/near-infrared lymphatic imaging**	Visualization of superficial lymphatic pathways, qualitative drainage pattern, tracer progression/transit, and dermal backflow patterns where applicable.	Can directly test whether regional lymphatic transit differs before and after treatment or between clinically defined exposure groups.	Invasive dye administration is required; imaging depth is limited and protocols for facial injectable research are not yet standardized. Findings should not be equated automatically with clinical lymphatic dysfunction.
**High-frequency ultrasound (HFUS)**	Filler location, depth, distribution, thickness, residual material, edema, nodules, tissue echogenicity, and relationships to adjacent vessels and soft-tissue layers.	Can quantify structural exposure and filler burden, distinguish material from edema or other tissue findings, and provide anatomical context for mechanical or lymphatic measurements.	Ultrasound appearance varies by product, device frequency, operator, timing, and tissue plane; HFUS does not by itself measure mechanotransduction or lymphatic function.

## Data Availability

No new data were generated or analyzed in support of this review.
